# Synergistic Inhibition of Pro-Inflammatory Pathways by Ginger and Turmeric Extracts in RAW 264.7 Cells

**DOI:** 10.3389/fphar.2022.818166

**Published:** 2022-05-19

**Authors:** Xian Zhou, Gerald Münch, Hans Wohlmuth, Sualiha Afzal, Ming-Hui (Tim) Kao, Ahmad Al-Khazaleh, Mitchell Low, David Leach, Chun Guang Li

**Affiliations:** ^1^ NICM Health Research Institute, Western Sydney University, Westmead, NSW, Australia; ^2^ School of Medicine, Western Sydney University, Campbelltown, NSW, Australia; ^3^ Integria Healthcare, Eight Mile Plains, QLD, Australia; ^4^ School of Chemistry and Molecular Biosciences, The University of Queensland, Brisbane, QLD, Australia

**Keywords:** ginger, turmeric, synergy, MAPK, TLR4-TRAF6-NFκB, miR-155-5p, cytokine array

## Abstract

Synergy plays a prominent role in herbal medicines to increase potency and widen the therapeutic windows. The mechanism of synergy in herbal medicines is often associated with multi-targeted behavior and complex signaling pathways which are challenging to elucidate. This study aims to investigate the synergistic mechanism of a combination (GT) of ginger (G) and turmeric (T) extracts by exploring the modulatory activity in lipopolysaccharides (LPS)-induced inflammatory pathways and key molecular targets. A Bioplex ProTM mouse cytokine 23-plex assay was utilized to assess the broad anti-cytokine activity of GT in LPS and interferon (IFN)-ɣ (both at 50 ng/mL)-activated RAW 264.7 cells. The inhibitory effects of individual and combined G and T on major proinflammatory mediators including nitric oxide (NO), tumor necrosis factor (TNF) and interleukin (IL)-6 were tested using Griess reagents and ELISA assays, respectively. Immunofluorescent staining and Western blot were used to investigate the modulatory effect of GT on key proteins in the LPS/TLR4 signaling transduction. The regulation of murine microRNA miR-155-5p was tested using real-time PCR. The IC_50_ value and combination index (CI) values were used to demonstrate potency and synergistic interaction, respectively. GT synergistically attenuated a range of pro-inflammatory mediators including inducible NO, major cytokines (TNF and IL-6) and secondary inflammatory cytokines (GM-CSF and MCP-1). GT significantly inhibited LPS-induced NF-kB p65 translocation, the activation of TLR4, TRAF6, and phosphorylation of JNK and c-JUN. Moreover, the suppressive effect of GT on each of the protein targets in this axis was stronger than that of the individual components. Real-time PCR analysis showed that GT suppressed miR-155-5p to a greater extent than G or T alone in LPS-stimulated cells. Our study demonstrates the synergistic mechanism of GT in downregulating LPS-induced proinflammatory pathways at the miRNA and protein levels. Our results establish a scientific basis for the combined application of G and T as an advanced therapeutic candidate in inflammatory diseases with broad and synergistic anti-inflammatory activity and multi-targeted mechanisms.

## Introduction

Inflammation is a physiological response by which the body attempts to counteract invading pathogens and injury (Hersh et al., 1998). Inflammation is associated with most chronic diseases including heart disease, diabetes, Alzheimer’s, arthritis and cancer, and is the product of complex series of responses triggered by the immune system (He et al., 2015). In the recent coronavirus disease (COVID-19) outbreak, many clinical observations suggested that systematic hyperinflammation with excessively elevated cytokine levels is the main driver of severity and mortality (Bhaskar et al., 2020; Webb et al., 2020). Although inflammation has been studied for decades, there has been little change in pharmacotherapy. Commonly used medications to manage inflammation include steroids and nonsteroidal anti-inflammatory drugs. However, these drugs are not suitable for long-term use as they are often associated with cardiovascular risks and gastrointestinal side effects (Meek et al., 2010; Solomon et al., 2017; Liu and Wu, 2019). In addition, the efficacy of nonsteroidal anti-inflammatory drugs against systematic inflammation such as cytokine storm and sepsis is limited partly due to their single-target action (Tisoncik et al., 2012; Abdin et al., 2020). Thus, researchers are still actively searching for broad anti-inflammatory drugs with a wide therapeutic window and multi-targeted actions to manage chronic and systematic inflammation.

Extensive research has shown that inflammation is associated with a complex cascade of signaling pathways, which presents a significant challenge for drug development (Van Dyke and Kornman, 2008). Lipopolysaccharides (LPS) is a principal component of the outer membrane of Gram-negative bacteria which triggers the production of various pro-inflammatory cytokines and systematic inflammation. The Toll-like receptor-4 (TLR-4), a key pattern recognition receptor, recognizes and binds LPS which contacts down-stream tumor necrosis factor receptor (TNFR)-associated factor 6 (TRAF6), then activates mitogen-activated protein kinases (MAPKs) and nuclear transcription factor-kappa B (NF-κB). The transcription of NF-κB is central to the mediation of the innate immune response leading to inflammation that induces the expression of downstream pro-inflammatory mediators and cytokines including inducible nitric oxide synthase (iNOS), interleukin (IL)-6, and tumor necrosis factor (TNF). In addition, recent research findings showed that signaling by TLRs not only induces downstream proinflammatory proteins but also provokes the activation of numerous miRNAs including miR-155, miR-146a, and miR-21 (O'Connell et al., 2012). In particular, recent findings have shown that miR-155, one of the proinflammatory miRNAs, precisely regulated the feedback loop of NF-κB to promote the immune response (Liu et al., 2020; O'Connell et al., 2012).

Natural products or derived bioactive compounds have formed “the backbone of modern pharmacopoeias” (Jia and Zhao, 2009). Enormous effort has been devoted to searching for drug candidates from natural sources to target inflammation and related conditions. For example, aspirin, first derived from natural salicylates isolated from willow bark, has been used extensively to relieve pain and inflammation. Notably, some phytomedicines have been shown to have greater therapeutic effects when used in combination than when used individually. The concept of the combined effect of several agents being greater than the sum of the effects of the individual agents is known as synergy (Zhou et al., 2016b). Nowadays, synergistic combination therapy has been widely accepted as a better and practical treatment strategy for complex diseases that need a systematic approach such as inflammatory diseases (Saw et al., 2010; Ramachandran et al., 2017; Le-Le et al., 2018; Zhang et al., 2019). The concept of synergy has long been embedded in traditional herbal formulations which rely on the positive interactions between different ingredients to achieve a multi-target effect and desired clinical outcome (Yang et al., 2014). Due to the complex chemical nature of herbal medicines, determining synergy within a herbal formulation usually starts with its major herbal ingredients, i.e. a pair of herbs, to identify the key contributing ingredients and the optimal ratio. In addition, several mathematical models such as the combination index (CI) and isobologram method, both of which can differentiate between synergistic and additive effects, have been extensively used to quantify the true synergistic interaction in phytomedicines, (Zhou et al., 2016b). For instance, our previous study applied the CI and isobologram models to identify synergistic interaction in a commonly used pair of herbs, *Salvia miltiorrhiza* and *Panax notoginseng*, in reducing inflammation, endothelial apoptosis and promoting angiogenesis (Zhou et al., 2016a; Zhou et al., 2017; Zhou et al., 2019).

Ginger (*Zingiber officinale* Roscoe, G) and turmeric (*Curcuma longa* L., T), both of the Zingiberaceae (ginger) family, are used extensively as both spices and phytomedicines. Numerous clinical and preclinical studies have shown their individual effectiveness in aiding inflammatory conditions including pain, arthritis, respiratory and gastrointestinal disorders (Habib et al., 2008; Terry et al., 2011; Kubra and Rao, 2012; Haniadka et al., 2013; Daily et al., 2016; Stoyell et al., 2016; Ebrahimzadeh Attari et al., 2018). An *in vivo* study reported that combined therapy of ginger and turmeric extracts (3,000 mg/100 ml, 1:1) exhibited a more significant effect in modulating lipid levels (triglycerides, cholesterol, high-density lipoprotein, low-density lipoprotein and very-low-density lipoprotein) than the components alone, in diabetic-dyslipidemic rats (Hussain et al., 2018). A mixture of ginger and turmeric powder in water (400 mg/kg body weight, 1:1 *w/w*), tested in a rat model of human rheumatoid arthritis, exerted greater effects (*p*<0.05–0.001) in reducing systematic inflammation in comparison to indomethacin (1.0 mg/kg body weight) (Ramadan and El-Menshawy, 2013). Although synergy has been demonstrated in these and other studies, little is known about how synergy occurs at the molecular level, in particular with respect to signaling pathways. This study aimed to examine the synergistic mechanism of G and T extracts *via* broad anti-inflammatory actions, enhanced efficacy and multi-targeted behavior in the LPS-induced inflammatory pathway. Understanding the relevant molecular mechanism may help to elucidate the foundation for their combined use in systemic inflammation and inflammatory-based conditions.

## Materials and Methods

### Preparation of Herbal Samples

Authenticated ginger root powder and turmeric extract [standardized ethyl acetate extract with enriched curcumin, demethyxocurcumin and bisdemethyxocurcumin, drug-extract ratio 25:1 (produced by Sami Labs Ltd., Bangalore, India, lot no. E17E041)] were obtained from Integria Healthcare Australia. The crude ginger root powder (over 100 g) was extracted with 90% ethanol (Chem-supply, cat no. EA043-20L-P) in 1 L by sonication (Power sonic 420, Thermoline Scientific, Australia) with the maximum volume under the room temperature for 30 min and 3 times. The supernatant was collected and filtered. Then the supernatant was dried by the rotary evaporator (Rotavapor R-215 coupled with vacuum controller v-850, BUCHI, Switzerland) and freeze drier (CHRIST, John Morris Scientific, Australia) overnight to obtain the dried extract. As per the product specification, the powdered extract of turmeric contained curcumin C) 80.2%, demethyxocurcumin D) 17.3% and bisdemethyxocurcumin B) 2.5% as determined by the high-performance liquid chromatography (Shimadzu, Australia). It was dissolved in 100% ethyl acetate (Sigma, cat. no. 34858-2L) and mixed by sonication. The supernatant was filtered and collected by rotary evaporation to remove the excipients. Finally, the extracts of G and T were dried by the freeze drier and stored at −20°C until use.

The GT combination was prepared by mixing the same concentration (50 mg/ml) of G and T in dimethyl sulfoxide (Sigma-Aldrich, Australia, cat. no: D8418) in the ratio of 5:2 by weight [equivalent to 7:10 (*w/w*) on the dried, crude rhizome basis]. The chemical profile of G and T were analysed by HPLC (Shimadzu, Australia) with established methods (Lee et al., 2007). The marker compounds in G and T were purchased from BioPurify Phytochemicals, China, including 6-gingerol (cat. no. BP0092), 8-gingerol (cat. no. BP0108), 10-gingerol (cat. no. BP0013), 6-shogaol (cat. no. BP0095), 8-shogaol (cat. no. BP1770), 10-shogaol (cat. no. BP1771), curcumin (cat. no. BP0421), bisdemethoxycurcumin (cat. no. BP0278), demethoxycurcumin (cat. no. BP0472). The amounts of 6-gingerol, 8-gingerol, 10-gingerol, 6-shogaol, 8-shogaol, 10-shogaol in G were 69.57 ± 0.16 mg/g, 10.43 ± 0.23 mg/g, 19.62 ± 0.63 mg/g, 7.48 ± 0.19 mg/g, 1.56 ± 0.03 mg/g, 2.30 ± 0.03 mg/g respectively, accounting for 11.10 ± 0.13% of the total weight. The amounts of curcumin, desmethoxycurcumin and bisdesmethoxycurcumin were 751.76 ± 101.45, 14.77 ± 3.63, 156.15 ± 26.24 respectively, accounting for 92.27 ± 13.13% of the total weight. The chemical fingerprints of G and T are shown in Supplementary Material S1.

### Cell Culture

The murine RAW 264.7 macrophages cells were cultured at 37°C in DMEM (Lonza, Australia, cat. no. 181563) supplemented with 5% foetal bovine serum (FBS) (Life Technologies, Australia, cat. no. 10437028), and 1% penicillin-streptomycin (Life Technologies, Australia, cat. no. 10378016) in a humidified atmosphere containing 5% CO_2_ and 95% air.

### Multi-Plex Assay

The inhibitory effects of individual and combined G and T were tested in a panel of 23 inflammatory mediators using a Bioplex ProTM mouse cytokine 23-plex kit (Bio-Rad, Australia, cat. no. #M60009RDPD) in RAW 264.7 cells. Briefly, RAW 264.7 cells were seeded at the density of 1 × 10^6^ in a 96-well cell culture plate (Corning^®^ Costar^®^, Sigma, Australia, cat. no. CLS3799) with 100 μL per well and placed in the incubator overnight. Then the cells were treated with G, T or GT extracts (50 μg/ml) with serial dilutions for 2 h before the stimulation with LPS (50 ng/ml, Sigma-Aldrich, Australia, cat. no. L4130) and interferon (IFN)-γ (50 ng/ml, Biolegend, Australia, cat. no. 575304) for another 16 h. The cell supernatant was then collected into another 96-well plate and centrifuged at 500 g for 5 min to remove the cell debris. The supernatant was then subjected to the Bioplex assay followed by the manufacturer’s protocol.

The 23 inflammatory mediators included pro-inflammatory cytokines: TNF (Zhang and An, 2007), interleukin–1a (IL-1a) (Thermo Fisher Scientific, 2022), IL-1b (Zhang and An, 2007), IL-6 (Zhang and An, 2007), IL-12 (Thermo Fisher Scientific, 2022), IL-17 (Thermo Fisher Scientific, 2022), IFN-ɣ (Thermo Fisher Scientific, 2022) and IL-12 (p40/p70) (Thermo Fisher Scientific, 2022); adaptive immunity: IL-2, IL-3, IL-5 and IL-9 (Thermo Fisher Scientific, 2022); chemokines: RANTES (Zhang and An, 2007), granulocyte colony-stimulating factor (G-CSF) (Thermo Fisher Scientific, 2022), GM-CSF (Thermo Fisher Scientific, 2022), KC (IL-8) (Zhang and An, 2007), MCP-1 (Zhang and An, 2007), MIP-1a/1b (Zhang and An, 2007) and eotaxin (Rothenberg, 1999). The anti-inflammatory cytokines included IL-3 (SinoBiological, 2022), IL-4 (Zhang and An, 2007; Chatterjee et al., 2014) and IL-10 (Zhang and An, 2007) and IL-13 (Thermo Fisher Scientific, 2022). The modulation of these cytokines after the stimulation of LPS and IFN-ɣ was analysed by unpaired t-test using GraphPad Prism 5.

### NO, TNF and IL-6 ELISA Assay

The RAW 264.7 cells (1 × 10^6^ cells/ml) were seeded in the 96 well plates (Corning^®^ Costar^®^, Sigma, Australia, cat. no. CLS3799) overnight until confluency. The cells were then treated with G, T, GT at 50 μg/ml or vehicle (0.1% DMSO) with serial dilutions for 2 h and followed by the stimulation of LPS (50 ng/ml, Sigma-Aldrich, Australia, cat. no. L4130) and IFN-ɣ (50 ng/ml, Peprotech, Australia, cat. no. 315–05) for another 16 h. The supernatants (50 μL) from RAW 264.7 cells were then collected and mixed with 25 μL of 1% sulfanilamide (Sigma-Aldrich, Australia, cat. no. S9251) in 5% phosphoric acid (Sigma-Aldrich, Australia, cat. no. 345245) and 25 μL of 0.1% N-1-naphthylethylenediamine dihydrochloride (Sigma-Aldrich, Australia, cat. no. 222488) in Milli-Q water (Milli-Q Advantage A10 System, Millipore Corporation, Germany). Sodium Nitrite (Sigma-Aldrich, Australia, cat. no. 67398, 0–10 μg/ml) was used as the standard in the NO assay. The absorbance was measured at 540 nm using the microplate reader (BMG LABTECH FLUOstar OPTIMA, Mount Eliza, Victoria, Australia).

After the stimulation of the LPS and IFN-ɣ, the cell supernatants were diluted by 100 times and subjected to the TNF and IL-6 ELISA assays which were conducted by the commercial ELISA kits (Lonza, Australia, cat. no. 900-K54; 900-K50) according to the manufacturer’s instructions. The standard murine TNF (0–100 pg/ml) and murine standard IL-6 (0–4,000 pg/ml) from the kits were used for the calibration curves. The absorbance was measured at 410 nm using the microplate reader (BMG LABTECH FLUOstar OPTIMA, Mount Eliza, Victoria, Australia).

### Expression of iNOS and NF-κB Nuclear Translocation

The expressions of iNOS and NF-κB nuclear translocation were assessed using confocal fluorescence microscopy. RAW 264.7 cells were plated in the 8 well Nunc™ Lab-Tek™ II Chamber Slide™ System (Thermo Fisher Scientific, Australia, cat. no. 154534) at 20,000 cells/well, incubated overnight, and then co-incubated with G, T, GT at 5 or 10 μg/ml or vehicle (0.1% DMSO) in DMEM serum free medium for 1 h prior to the stimulation of LPS (1 μg/ml) for 30 min. Cells were then washed with ice cold phosphate-buffered saline (PBS) buffer (Sigma-Aldrich, Australia, cat. no. P4417-100TAB) and fixed using 2% paraformaldehyde (Cell Signaling Technologies, United States, cat. no. 12606S) for 30 min at room temperature. Triton X 100 (0.1%, Thermo Fisher Scientific, Australia, cat. no. HFH10) was used to permeabilize the cells for 20 min. Cells were then washed again with PBS (three times) and blocked using 3% of bovine serum albumin (Bovogen Biologicals, Australia, cat. no. BSAS 1.0) for 1 h followed by overnight incubation at 4°C with rabbit anti-iNOS (Cell signaling technology, Australia, cat. no. 13120S, 1:200) or mouse anti-p65 NF-κB antibody (Santacruz, Australia, cat. no. sc-166748, 1:200). Cells were washed again with PBS and incubated with the donkey anti-rabbit IgG conjugated with Alexa Fluor 594 (red dye, Thermo Fisher Scientific, cat. no. A-32754, 1:1000) or donkey anti-mouse IgG conjugated with Alexa Fluor 488 (green dye, Thermo Fisher Scientific, cat. no. A-32766, 1:1000) for 1 h in the dark room at room temperature. After washing with PBS, the chambers were removed and the anti-fade mounting media with DAPI solution (blue color, Sigma-Aldrich, Australia cat. no. MBD0020) was added before capturing images with the Inverted Leica TCS SP5 laser scanning confocal microscope (School of Medicine, Western Sydney University, Australia). The fluorescent intensity was quantified and analysed using ImageJ. Samples were run in triplicate and over 10 representative cells were chosen at random in each treatment well to assess iNOS expression and NF-κB location. The results were processed as the total cell fluorescence (CTCF) corrected against the background (no fluoresce).

### Immunoblotting Analysis

RAW 264.7 cells were grown in T75 cell flasks (SARSTEDT, Australia, cat. no. 83.3911.300) until confluence. Cells were then treated with G, T, GT at 10 μg/ml or media with vehicle (0.1% DMSO) for 1 h before being activated with LPS (1 μg/ml). After the incubation for 40 min or 24 h, cell pellets were harvested by centrifugation at 500 g for 5 min. The cell pellets were mixed with 100 μL of the ice-cold RIPA lysis buffer (Santa Cruz Biotechnology, Australia, cat. no. sc-24948A) and 0.1% of the protease/phosphatase inhibitor cocktail (Cell Signaling Technologies, United States, cat. no. 5872S) on ice for 30 min (vortex every 5–10 min). Then the cell lysates were centrifuged at 16000 g for 5 min. The supernatant was collected and subjected to protein estimation by the Pierce™ BCA Protein Assay Kit (Thermo Fisher Scientific, Australia, cat. no. 23225). The total proteins from each sample at 10 mg/ml were separated by the SDS-PAGE electrophoresis (PowerPac HC, BIORAD, Australia) under 90V for 1 h in the 10% Mini-PROTEAN TGX Precast Protein Gels (10-well, BIORAD, Australia, cat. no. 4561034), and then the protein was transferred by the iBlot 2 Dry Blotting System (Thermo Fisher Scientific, Australia, cat. no. IB21001) using the IBLOT PVDF gel transfer stacks regular kit (Thermo Fisher Scientific, Australia, cat. no. IB24001) at 10V for 3 min. The PVDF membrane was then incubated with 3% BSA dissolved in PBS-T [PBS buffer plus 1% tween 20 (Thermo Fisher Scientific, Australia, cat. no. 003005)] for 60 min at room temperature. The membranes were incubated with anti-TLR4 (1:1000, cat. no. 14358), anti-TRAF6 (1:1000, cat. no. 67591), anti-phospho-p38 (1:1000, cat. no. 9216S), anti-p38 (1:1000, cat. no. 8690S), anti-phospho JNK (1:1000, cat. no. 4668), anti-JNK (1:1000, cat. no. 9252), anti-phospho-cJUN (1:1000, cat. no. 32700), anti-cJUN (1:1000, cat. no. 9165) overnight at 4°C. The membranes were washed by PBST buffer 3 times and soaked in anti-rabbit conjugated with horseradish peroxidase (1:1000, cat. no. 7074) or anti-mouse conjugated with horseradish peroxidase (1:1000, cat. no. 7076) for 1 h. All these antibodies were purchased from Cell Signaling Technology (United States). The immunoreactive bands on the membranes were visualized by adding 1 ml of the mixed reagents A and B from the Supersignal West Pico Plus ECL kit (Thermo Fisher Scientific, Australia, cat. no. 34577). Specific bands were analysed and the intensity was quantified using ImageJ software.

### qPCR Analysis

RAW 264.7 cells were seeded in the T25 cell culture flask (SARSTEDT, Australia, cat. no. 83.3910.300) with 10% FBS in the DMEM until confluency. The cells were treated with G, T, GT at 10 μg/ml or media with 0.1% DMSO for 2 h followed by the stimulation of LPS (1 μg/ml) for 16 h. The total RNA was obtained from the cells after the treatment and stimulation using the mirVana RNA isolation kit (Thermo Fisher Scientific, Australia, cat. no. AM1560). The quantity and purity of total RNA were determined by a nanodrop spectrophotometer (Implen, United States). The reverse transcription (RT) of approximately 10 ng of the total RNA was performed using the TaqMan™ MicroRNA Reverse Transcription Kit (Thermo Fisher Scientific, Australia, cat. no. 4366596) to synthesise cDNA using the Eppendorf^®^ Mastercycler^®^ Pro Thermal Cyclers (Eppendorf, Germany). The performance was based on the instruction in the kit as follows: 16.0°C for 30 min, 42.0°C for 30 min, 85°C for 5 min and stopped at 4°C. The cDNA samples were then prepared with the TaqMan™ MicroRNA Assay (Thermo Fisher Scientific, Australia, cat. no. 4427975) before subjecting to the stem-loop RT-PCR (Mx3000/Mx3005P Real-Time PCR system, Agilent, Australia). Then the qPCR was performed after the TaqMan™ MicroRNA Assay using TaqMan™ Fast Advanced Master Mix (Thermo Fisher Scientific, Australia, cat. no. 4444557). Each 20 μL reaction contained 10 μL SYBR Premix, 1 μl each primer, 1.33 μL cDNA and 7.67 RNase-free dH2O. The primer sequences for miR155-5p (cat. no. 4464066, assay ID: 002571) and internal controls using U6 small nuclear RNA (cat. no. 4464084, assay ID: 001973) were proprietary to Thermo Fisher Scientific (Australia). Amplification conditions for miR-155-5p detection were set to heat activation at 95°C for 20 s followed by 40 cycles of denaturation at 95°C for 3 s, annealing at 60°C for 30 s. Data were then analysed by the comparative CT method (ΔΔCT Method) normalized against U6 snRNA levels to unveil the expression fold change between the treatments. All reactions were conducted from three individual experiments.

### Determination of Synergistic, Additive or Antagonistic Interactions

The synergistic/antagonistic interaction between G and T was determined using the CompuSyn software 2.0 (Biosoft, United States). Briefly, the values of the concentration and the correspondent responses of G, T and GT in the measurements of each cytokine in Multi-plex assay, NO, TNF and IL-6 ELISA assays were input to the CompuSyn software 2.0 respectively for the isobologram and CI analysis of synergy. The program then generated the CI-fraction affected (Fa) curve, isobologram figure, and the CI values at all Fa values. In the CI-Fa curve, Fa refers to the default effect level set between 0,1. In our study, Fa presented the suppressive responses on NO, IL-6 or TNF from 0% to 100%. The CI values were used to demonstrate the interaction, with CI < 1 representing synergistic interaction, CI = 1 representing no interaction (additive effect) and CI > 1 representing antagonistic interaction (Zhou et al., 2016b). The isobologram graphics were used to show synergy at three set concentrations (Fa = 0.5, Fa = 0.75 and Fa = 0.9).

### Statistical Analysis

All data were expressed as mean ± SD (*n* ≥ 3) and analysed by GraphPad Prism 8. One-way analysis of variance was used to assess the statistical difference among groups. Values of *p* < 0.05 were considered statistically significant.

## Results

### Broad and Synergistic Anti-inflammatory Activities of GT in LPS-Induced RAW 264.7 Cells

To explore the broad anti-inflammatory activities of G, T or GT, a multi-plex assay was utilized to examine the changes of various cytokines after treatment in LPS and IFN-γ-induced RAW 264.7 cells. As shown in Table 1, the stimulation by LPS and IFN-ɣ for 24 h led to a significant up-regulation of all of the tested pro-inflammatory cytokines, secondary cytokines, and chemokines to the range of 6.06 pg/ml to 97 ng/ml, except for macrophage inflammatory protein (MIP)-1b. LPS and IFN-ɣ also enhanced the production of anti-inflammatory cytokines such as IL-10 and IL-13.

**TABLE 1:** LPS and IFN-ɣ significantly induced multiple cytokines in RAW 264.7 cells. Data are shown as mean concentration (pg/ml) ± STD and fold change from duplicates.

Inflammatory mediators		Concentration in RAW 264.7 macrophages (pg/ml)		
		Non-activated	LPS and IFN-ɣ-activated	Upregulation (fold)
Pro-inflammatory	IL-1a (53)	33.12 ± 0.06	666.61 ± 58.15	20.25**
	IL-1b (19)	10.13 ± 0.59	206.33 ± 29.51	20.37**
	IL-6 (38)	1512.25 ± 131.88	25416 ± 321.03	16.81**
	IFN-ɣ (34)	11.90 ± 0.42	29.76 ± 0.61	2.50***
	IL-12 (p40) (76)	17.96 ± 0.60	527.99 ± 40.16	29.41**
	IL-12 (P70) (78)	45.28 ± 2.95	129.83 ± 1.12	2.87*
	TNF	1598.75 ± 240.06	24104.75 ± 127.63	15.08***
	IL-17 (72)	3.01 ± 0.06	7.37 ± 0.52	2.45*
Adaptive immunity	IL-2 (36)	5.99 ± 0.40	69.38 ± 5.02	11.59**
	IL-3 (18)	1.67 ± 0.05	6.06 ± 0.08	3.64**
	IL-5 (52)	3.91 ± 0.23	10.41 ± 0.07	2.66*
	IL-9 (33)	7.65 ± 0.53	18.36 ± 0.06	2.4*
Chemokines	Eotaxin (74)	16.58 ± 1.19	44.59 ± 1.87	2.69**
	RANTES	5141.16 ± 425.11	16178.34 ± 1007.06	3.14*
	G-CSF (54)	16228.64 ± 452.66	97776.79 ± 2777.44	6.02**
	GM-CSF (73)	34.88 ± 1.07	2231.43 ± 202.15	63.97***
	KC (57)	5.27 ± 0.35	29.79 ± 0.91	5.65**
	MCP-1	1753.59 ± 139.79	45061.93 ± 61.53	25.70***
	MIP-1a	18800.75 ± 78.13	23442.75 ± 92.98	1.25*
	MIP-1b	23467.5 ± 53.03	23496.5 ± 16.26	1.00
Anti-inflammatory	IL-4 (39)	4.65 ± 0.00	7.57 ± 0.24	1.63*
	IL-10 (56)	19.63 ± 1.30	82.12 ± 7.45	4.18*
	IL-13 (37)	496.02 ± 14.91	1044.12 ± 5.27	2.10*

*p<0.05, **p<0.01, ***p<0.001 between non-activated and activated cells.

The effects of G, T and GT (1.625–50 μg/ml) for pro-inflammatory and secondary inflammatory mediators are shown in Table 2. G did not show a remarkable inhibitory effect on most cytokines, however moderate effects were detected for secondary inflammatory mediators and chemokines including granulocyte-macrophage colony-stimulating factor (GM-CSF), keratinocyte-derived cytokine (KC) and monocyte chemoattractant protein-1 (MCP-1) with IC_50_ values ranging from 5.97 to 16.69 μg/ml. In contrast, T showed potent inhibition of all of the cytokines (except MIP-1b) with the IC_50_ values ranging from 2.15 to 7.26 μg/ml. The inhibitory effect of GT on these mediators was generally stronger or comparable to that of T alone (excluding G-CSF and IL-17), with IC_50_ ranging from 3.84 to 22.50 μg/ml. Moreover, the dosage of G and T in GT required to reach the same effect level was generally lower than that of the individual extracts, especially for G. For instance, to inhibit IL-1b by 50%, the concentrations of G or T were 22.34 and 3.26 μg/ml, respectively, whereas they were only 3.66 and 1.44 μg/ml, respectively, when combined in GT. This trend was found for all the cytokines tested, demonstrating the positive, synergistic interaction of G and T. All the observed anti-cytokine activities by G, T or GT were not induced by cytotoxicity (data not shown).

**TABLE 2:** Synergistic effects of G-T 5:2 in inhibiting LPS and IFN-γ-induced cytokines in RAW 264.7 cells. Data are shown as mean IC_50_ and CI values at IC_50_ from duplicates.

Inflammatory mediators		IC_50_ (µg/ml) of G and T as individual extracts		IC_50_ of GT (µg/ml)	Concentration of G and T in the IC_50_ value of GT (µg/ml)		CI of GT at IC_50_
		G	T	GT	G	T	
Pro-inflammatory	IL-1a (53)	51.26	2.84	4.45	3.18	1.27	0.69
	IL-1b (19)	22.34	3.26	5.04	3.6	1.44	0.60
	IL-6 (38)	858.35	7.26	11.49	8.21	3.28	0.46
	IFN-g (34)	107.95	2.89	6.29	4.49	1.80	0.66
	IL-12 (p40) (76)	ND	2.40	11.36	9.28	3.71	1.55
	IL-12 (P70) (78)	-	3.68	12.99	3.81	1.52	0.41
	TNF	449.64	5.28	5.33	9.91	3.97	0.77
	IL-17	ND	5.38	22.50	16.07	6.42	43.86
Adaptive immunity	IL-2	1.10	0.60	0.68	0.49	0.19	3.00
	IL-3	65.02	4.91	2.21	1.58	0.63	1.87
	IL-5 (52)	ND	3.00	7.96	5.69	2.27	0.76
	IL-9 (33)	3,806.18	3.58	7.49	5.35	2.14	0.60
	Eotaxin (74)	58.03	4.25	6.40	4.57	1.83	0.51
	RANTES	25331.4	3.17	13.88	8.11	3.25	1.02
	GM-CSF (73)	16.69	2.61	17.23	2.97	1.19	0.63
	G-CSF (54)	214.57	4.84	13.62	176.74	70.69	15.43
	KC (57)	9.77	2.15	4.16	3.11	1.24	0.90
	MCP-1	5.97	2.21	4.36	2.74	1.10	0.95
	MIP-1a	111.41	4.33	3.84	9.73	3.89	0.99

ND, not detected, MIP-1b IC_50_ calculations were not possible (NA) as MIP-1b was not stimulated in the model used.

The synergistic interactions of G and T in inhibiting each cytokine in the array were verified by the CI model using CompuSyn. Synergy was detected for most of the cytokines and chemokines with CI values ranging from 0.46 to 0.99. Only for IL-12 (p40), RANTES, G-CSF and IL-17 were GT found to have additive (CI = 1) or antagonistic effects, with observed CI values at 1.55, 1.02, 15.43 and 43.86 respectively.

In addition, the activity of G, T and GT in inhibiting the major proinflammatory mediators NO, TNF and IL-6 were specifically measured in ELISA assays on RAW 264.7 cells and their interactions were analysed in the CI model. In the presence of LPS (50 ng/ml) and IFN-ɣ (50 ng/ml), a significant amount of nitrite, TNF and IL-6 productions were detected and reached 2.07 ± 0.15 μg/ml (*vs.* blank at 0.041 ± 0.00 μg/ml), 64.80 ± 3.12 ng/ml (*vs.* blank at 2.06 ± 1.34 ng/ml), 24.61 ± 7.73 ng/ml (*vs.* blank at 0.19 ± 0.75 ng/ml) calculated against each calibration curve and dilution factors, in DMSO vehicle solutions, respectively.

**FIGURE 1:**
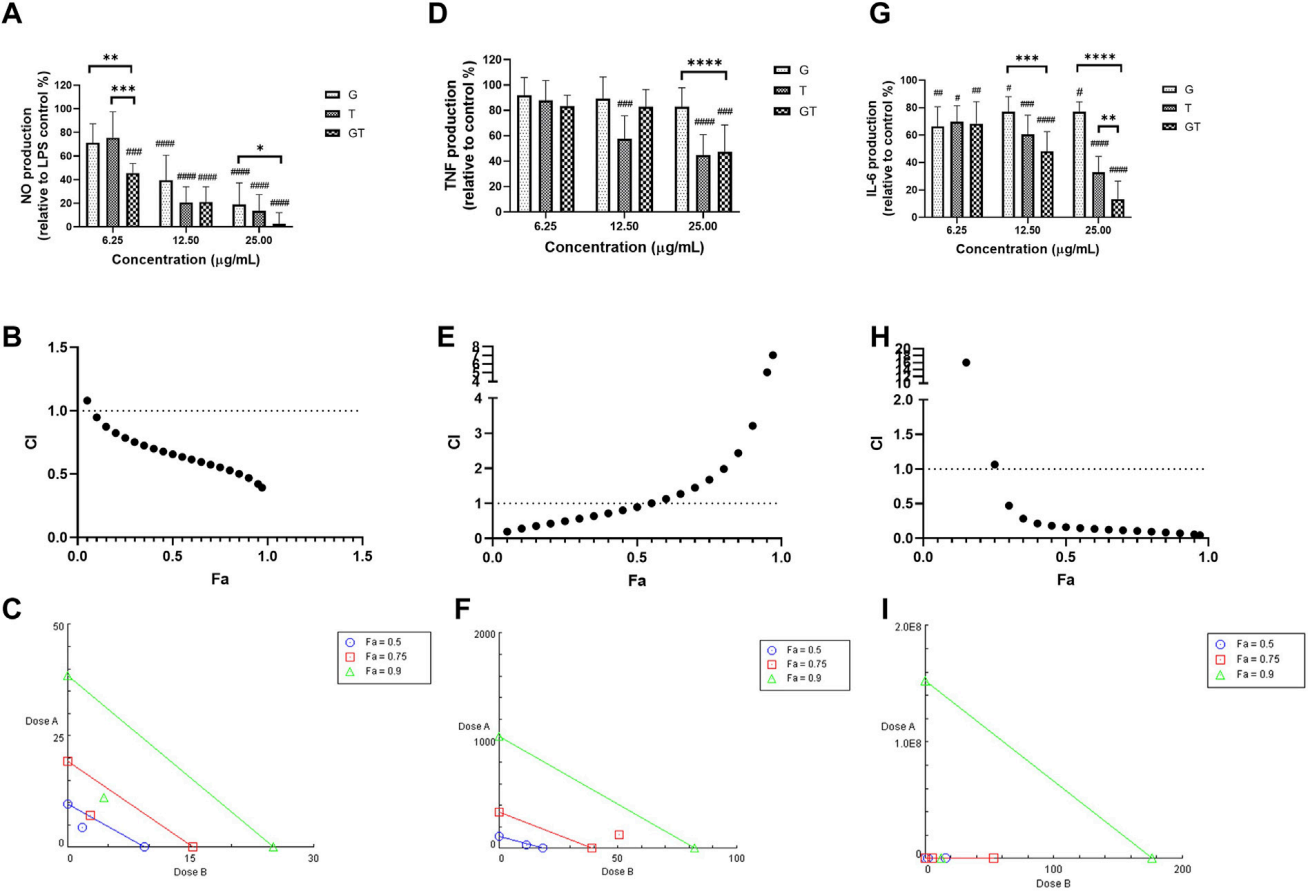
G, T and GT (all at 6.25, 12.5 and 25 μg/ml) dose-dependently inhibited NO **(A)**, TNF **(D)** and IL-6 **(G)** in LPS-induced RAW 264.7 cells (n≥3). The production of NO, TNF and IL-6 were expressed as percentage relative to LPS stimulation control (set as 100%). #*p*<0.05, ##*p*<0.01, ###*p*<0.001, ####*p*<0.0001 *vs.* LPS stimulation control. **p<0.01, ***p<0.001, ****p<0.0001 compared between GT to G or T at the same concentration level. The synergistic effects of GT were determined by the CI-Fa curves in reducing NO **(B)**, TNF **(E)** and IL-6 **(H)**. CI values represent the interaction in GT, with CI < 1, CI = 1 and CI > 1 referring to synergy, addition and antagonism, respectively. Fa on the X-axis is defined as the fraction effect level, and herein it refers to NO, TNF and IL-6 inhibitory effect, respectively. Isobologram analysis of GT in NO **(C)**, TNF **(F)** and IL-6 **(I)** when the default set of Fa values at 0.5, 0.75 and 0.9.

As shown in Figure 1A, all the extracts of G, T and GT (6.25, 12.5 and 25 μg/ml) showed dose-dependent effects against NO, and GT exhibited the greatest inhibitory effects at three tested concentrations. At the concentrations of 12.5 and 25 μg/ml, the inhibitory effects of all three extracts were significant (*p*<0.001) in comparison to LPS stimulation only (set at 100%). Moreover, GT showed a significantly greater NO inhibition at 6.25 μg/ml compared to that of G (*p*<0.01) and T (*p*<0.001). At 25 μg/ml, the No reduction of GT was greater than that of G only (*p*<0.05). A strong synergy of GT was observed in inhibiting NO as shown by the CI-Fa curve (Figure 1B), with CI values ranging from 0.39 to 0.94 when the Fa was above 0.1 (10%–100% NO inhibitory effect). The isobologram in Figure 1C also supported the observed synergy of GT in reducing LPS-stimulated NO when Fa values were at 0.9, 0.75 and 0.5 (representing 90, 75 and 50% of the NO inhibition). In Figures 1D–G did not show an evident TNF inhibitory effect from 6.25 to 25 μg/ml, whereas T dose-dependently reduced the TNF production, with significant inhibitions shown at 12.5 and 25 μg/ml (both *p* < 0.001 *vs.* LPS = 100%). GT did not show an obvious reduction until the concentration reached 25 μg/ml (*p*<0.001 *vs.* LPS = 100%). At that concentration level, the TNF reduction by GT was greater than that of G (*p*<0.0001). A weak synergy of GT was observed in inhibiting TNF (Figure 1E) with CI values ranging from 0.19 to 0.90 when the Fa level was below 0.50 (less than 50% NO inhibition). The isobologram analysis in Figure 1F demonstrated an antagonistic effect when Fa values were at 0.9 and 0.75, whereas the interaction became additive towards synergy when Fa = 0.5. This finding was consistent with that of the CI-Fa curve. In Figure 1C, all the extracts of G, T and GT dose-dependently inhibited the IL-6 production at all three concentrations (all *p*<0.05 *vs.* LPS = 100%), and GT showed significantly greater IL-6 inhibition at 12.5 μg/ml than that of G (*p*<0.001). At 25 μg/ml, the IL-6 inhibition by GT was greater than that of G (*p* < 0.0001) and T (*p* < 0.01). GT also exhibited a strong synergistic effect in reducing IL-6 (Figure 1H) with CI ranging from 0.05 to 0.47 at Fa levels of 0.3–0.97. The isobologram (Figure 1I) analysis showed a very strong synergy when Fa≤0.9 which was consistent with that of the CI-Fa curve. All the observed NO, TNF and IL-6 inhibitory activities by G, T or GT were not induced by cytotoxicity (data not shown).

### GT Further Suppressed LPS-Induced Elevated iNOS Expression, NF-κB Nuclear Translocation and miR-155-5p Levels in RAW 264.7 Cells

To investigate the synergistic mechanism of GT in inhibiting LPS-induced pro-inflammatory events, the key protein and miRNA targets including iNOS, NF-κB and miR-155-5p with or without G, T and GT treatments were examined.

**FIGURE 2:**
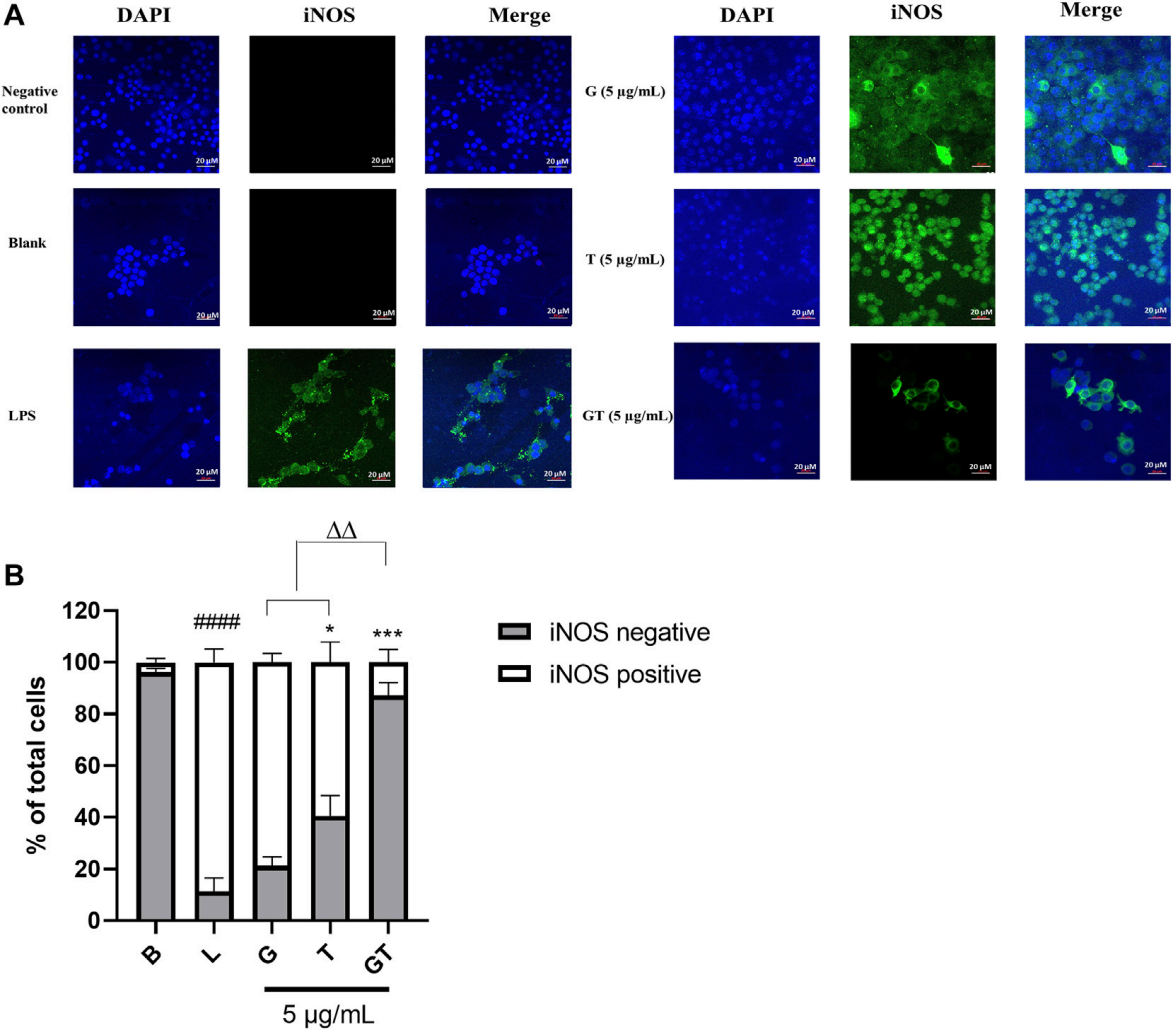
G, T and G-T 5:2 inhibited LPS activated iNOS expression in the RAW 264.7 cells by immunofluorescence staining of iNOS **(A)** Representative images of iNOS green fluorescent expression were taken by using a confocal microscope with 40× magnification. Blue: DAPI in the nucleus, green: iNOS in the RAW 264.7 cells. **(B)** Quantitative analysis of iNOS positive and negative expressions based on the fluorescent staining. ####*p*<0.05 of iNOS positive compared with blank **(B)**. *p<0.05 of iNOS positive compared with LPS (L). ***p<0.001 of iNOS positive compared with L. ∆∆ *p*<0.01of iNOS positive of GT compared with G and T.

In LPS (1 μg/ml)-activated RAW 264.7 macrophages, the number of cells expressing iNOS green fluorescence was significantly higher than that of the blank control (Figures 2A,B, *p*<0.0001, LPS *vs.* Blank). The inhibitory effect of G (10 μg/ml) was not significant at 24 h post-treatment, although there was a tendency to decrease the number of iNOS positive, activated cells. A significant inhibitory activity was seen in T (10 μg/ml, *p*<0.5 *vs.* LPS), whereas G-T 5:2 showed the most prominent activity (10 μg/ml, *p*<0.01 *vs.* LPS; *p*<0.01 *vs.* G or T).

**FIGURE 3:**
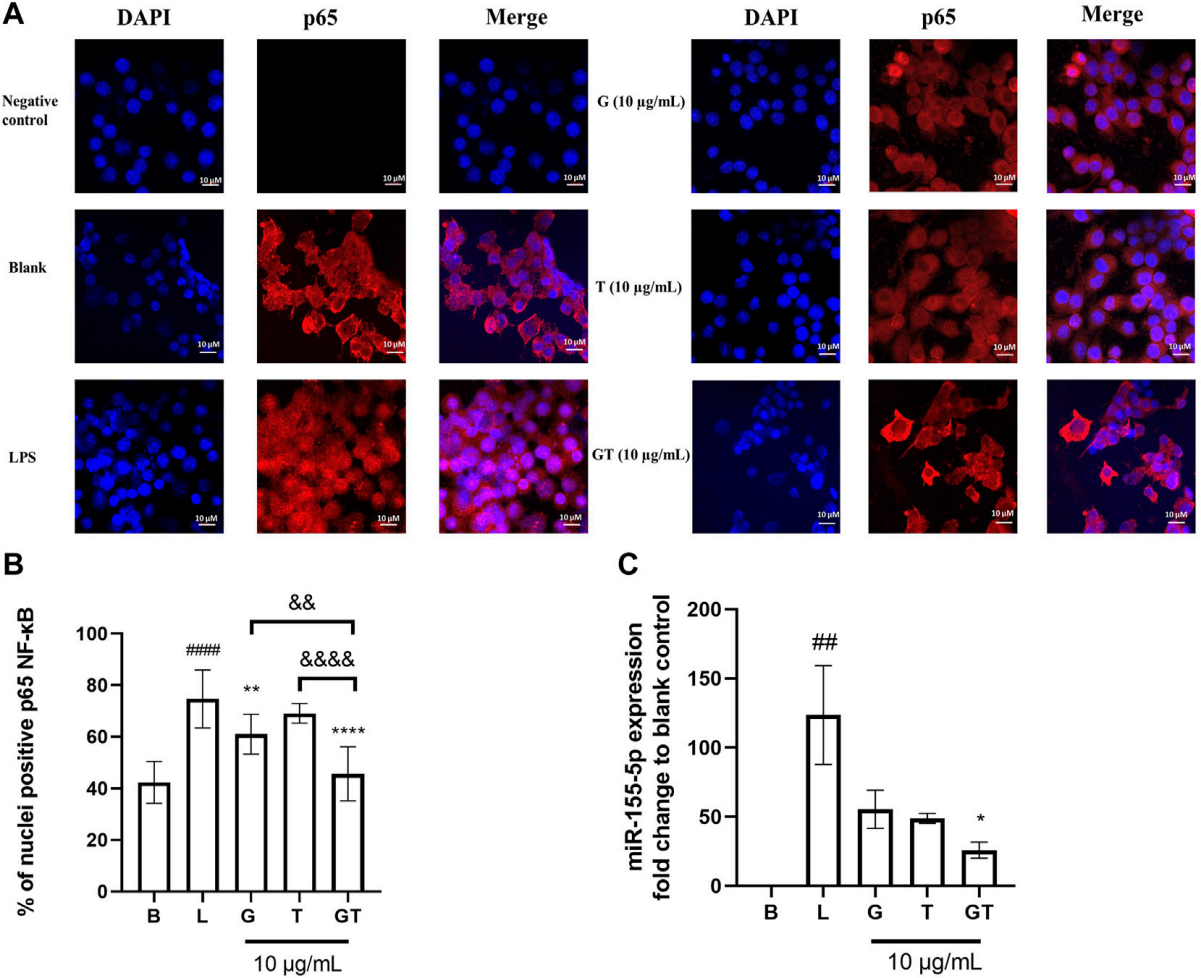
Down-regulated NF-κB translocation and miR-155-5p by G, T and GT **(A)** Representative images of G, T, and G-T 5:2 in inhibiting LPS activated NF-κB p65 translocation in the RAW 264.7 cells by immunofluorescence staining. Images (*n*≥5) were taken by using a confocal microscope with ×60 magnification. Blue: DAPI in the nucleus, red: NF-κB p65 in the RAW 264.7 cells **(B)** Quantification of % of nuclei positive p65 of staining in unstimulated, LPS stimulated macrophages with and without various treatments. Data points represent the mean ± standard deviation from the analysis of 9 separate cells. ####*p* < 0.0001 LPS (L) vs. Blank **(B)**, **p<0.01 compared with L, ****p<0.0001 compared with L. ∆∆ *p*<0.01 GT vs. G, ∆∆∆∆ *p*<0.0001 GT vs. T **(C)** Averaged fold change of miR-155-5p treated with G, T or GT with the stimulation of LPS (*n* = 3). ##*p*<0.01 LPS (L) vs. Blank **(B)**, *p < 0.05 GT compared with L.

In the absence of LPS, NF-κB p65 was observed almost exclusively in the cytoplasm (fluor 594 red staining of p65 was expressed outside the nucleus and DAPI blue was expressed in the nucleus) (Figure 3A). The nuclear content of p65 increased dramatically following the stimulation of LPS (100 ng/ml) as indicated by the overlapping of the p65-fluor 594 red fluorescence with the DAPI blue staining in the nucleus. The statistical analysis in Figure 3B showed a significantly higher percentage of p65 in the nuclei after the stimulation of LPS (*p*<0.001) compared to that of control. The LPS induced translocation of p65 has been reduced by G, T and GT at 10 μg/ml. G showed significant suppressing effect with *p* < 0.01 and GT further strengthened this effect with *p*<0.0001 *vs.* LPS, *p*<0.01 *vs.* G and *p*<0.0001 *vs.* T.

Three individual experiments were conducted to examine the modulatory effects of GT on mmu-miR-155-5p. Figure 3C shows that the averaged ∆∆Ct value of LPS (1 μg/ml) was 6.78 ± 0.53, which resulted in a 123.62 ± 35.67 fold increase in mmu-miR-155-5p expression. G, T and GT all reduced the elevated expression of 155-5p to less than 50-fold. In particular, GT showed the greatest inhibitory effect with the fold change at 25.91 ± 9.98, and it was significantly lower than that of LPS (*p*<0.05), and markedly lower than that of G (55.46 ± 23.95) or T (48.88 ± 6.22).

### Regulation of TLR4-TRAF6-MAPK Signaling Pathways

The synergistic mechanism of GT in inhibiting pro-inflammatory mediators in the protein levels was further investigated in the expressions of key protein targets in the TLR4-TRAF-6-MAPK pathway.

**FIGURE 4:**
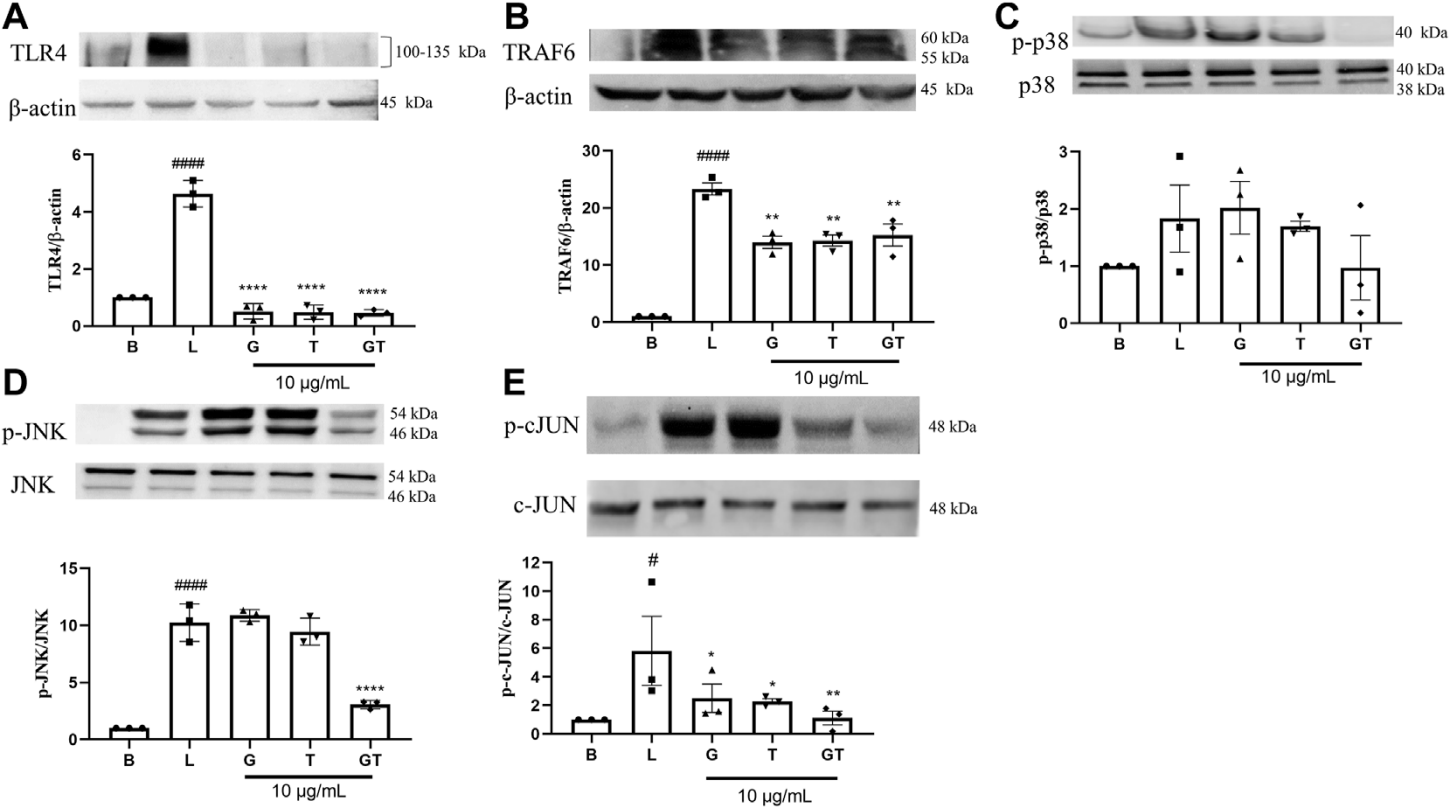
Down-regulatory effects of G, T and GT on the key protein targets in the TLR4-TRAF6-MAPK pathway. Cells were cultured in T75 cell flasks and were pretreated with G, T or GT 1 h prior to LPS (100 ng/ml) for various optimal time points. Protein expression levels of TLR4 **(A)**, TRAF-6 **(B)**, p-p38 **(C)**, p-JNK **(D)**, p-cJUN **(E)** were analyzed by western blot using whole protein extract. All results (*n* = 3) are expressed as the mean ± SEM, #*p*<0.05 vs. B; ####*p*<0.0001 vs. Blank **(B)**. *p<0.05, **p<0.01 vs. LPS (L).

**FIGURE 5:**
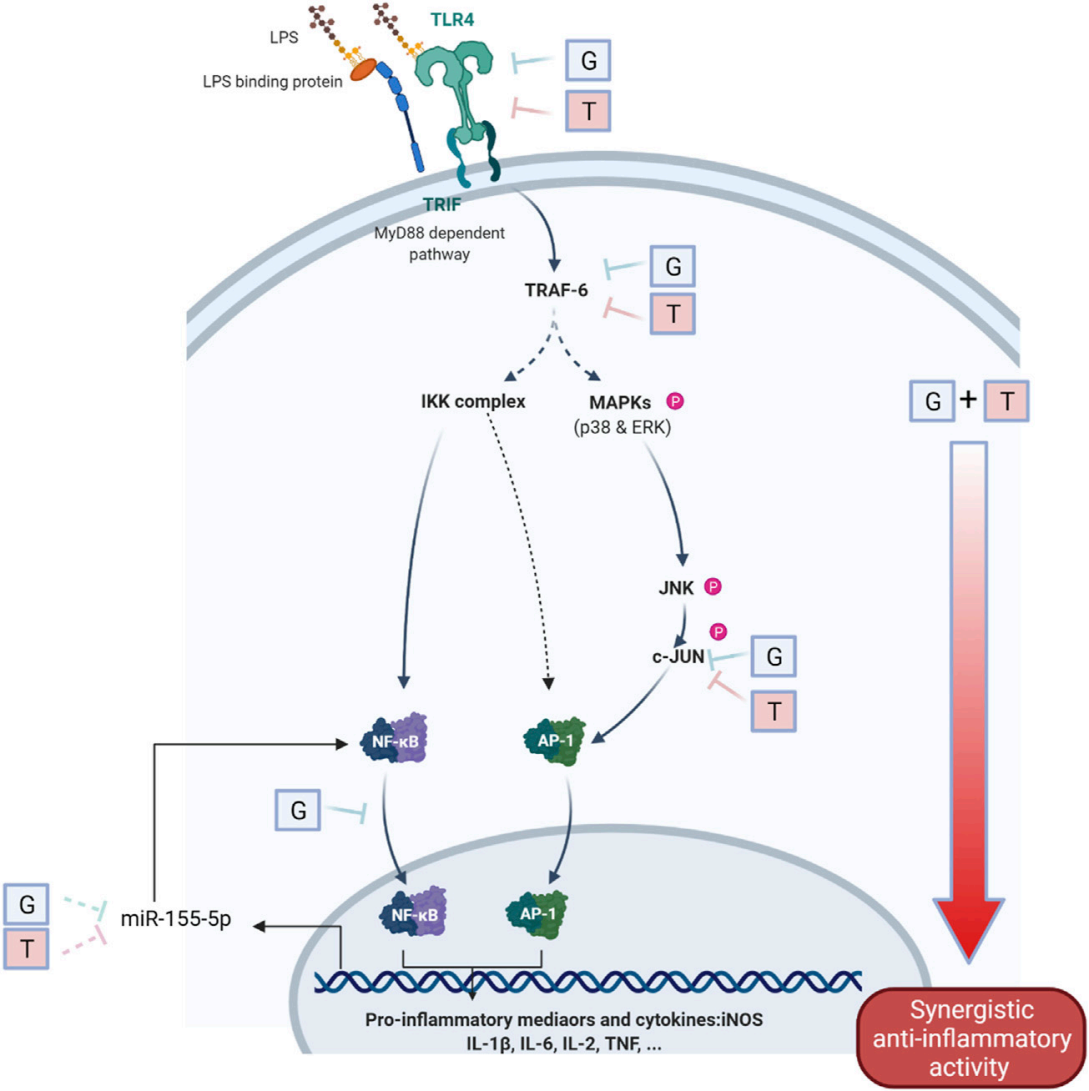
Schematic diagram of the interaction of G and T on LPS-induced proinflammatory pathway. The synergistically enhanced activity of GT in reducing proinflammatory mediators may attribute to the accumulated combined individual activities of G and T in TLR4, TRAF6 and further strengthened downregulation in p-JNK and p-c-JUN. The inhibitory effect on miR-155–5p may further emphasized the inhibition of pro-inflammatory *via* a negative NF-κB feedback loop.

In comparison to that of the untreated cells (Figure 4), the stimulation of LPS (1 μg/ml) led to upregulated expressions of TLR4 (*p*<0.0001), TRAF-6 (*p*<0.0001), p-p38/p38, pJNK/JNK (*p*<0.0001), and p-cJUN/cJUN (*p*<0.05) with fold increases of 4.63 ± 0.46, 23.31 ± 1.80, 1.83 ± 1.02, 10.25 ± 1.63, and 5.82 ± 4.18, respectively. G was shown to inhibit the increased fold change of TLR4/β-actin (*p* < 0.0001), TRAF-6/β-actin (*p* < 0.01) and p-cJUN/cJUN (*p*<0.05). Surprisingly, the highest inhibition of G was shown in TLR4. T also showed potent inhibition of TLR4/β-actin (*p*<0.0001), TRAF-6/β-actin (*p*<0.01) and p-cJUN/cJUN (*p*<0.05). The trend was found to be similar to that of G. GT showed comparable inhibitory effects to that of G and T on TLR4/β-actin (*p*<0.0001) and TRAF-6/b-actin (*p*<0.01). However, the inhibitory effects of GT became greater in comparison to G or T when it acted on the down-stream proteins of pJNK/JNK (*p*<0.0001) and pcJUN/cJUN (*p*<0.01). A Schematic diagram explaining the interaction of G and T on LPS-induced proinflammatory pathway is shown in Figure 5.

## Discussion

We have explored the broad anti-inflammatory effects of GT in reducing a range of cytokines in a synergistic manner as determined by the CI model, and investigated the detailed mechanistic action of both individual and combined G and T in regulating key proinflammatory mediators in LPS-provoked inflammatory response at the microRNA and protein levels. This is the first study to demonstrate the true synergistic interaction of G and T with broad anti-cytokine activity and demonstrate that the synergistic mechanism was associated with downregulation of the miRNA-155-5p and key proteins in the TLR4-TRAF6-MAPK and NF-κB pathways.

Overwhelming and uncontrolled inflammatory responses can lead to severe endotoxin shock and even death (Miyake, 2004). Such overwhelming inflammatory responses have been referred to as a cytokine storm and have been observed in many severe and deadly cases of inflammatory and infectious diseases including COVID-19 (Hu et al., 2021). Cytokine storm can be triggered by external stimuli such as viruses and bacteria, although the pathogenesis is quite complex (Ye et al., 2020). Several cytokines have been found to play a pivotal role in driving the excessive host inflammatory responses, including TNF, IL-6, IL-1β and IL-17A (Flierl et al., 2008; Leija-Martínez et al., 2020; Roshanravan et al., 2020).

The present study used both LPS and IFN-γ to trigger the multi-cytokine release from the RAW 264.7 cells, and quantified the cytokine levels simultaneously modulated by G, T or GT to ascertain their potential to inhibit a broad range of cytokines triggered by bacteria and viruses. Our data show a significant elevation of multiple cytokines in response to LPS and IFN-γ.

It is noticed that the effect of G was weak in inhibiting most of the cytokines (i.e., IL-6, TNF, IFN-γ and IL-9), while T showed a potent activity in reducing all tested cytokines. Despite the weak effect of G, this extract contributes to the synergistic, enhanced inhibitory effects of GT rather than diluting the effect of T. In addition, GT showed remarkable inhibition of all cytokines tested, including those not inhibited by G alone. Thus, our data highlights that G and T acted synergistically to enhance the overall activity.

As the Bioplex Pro™ mouse cytokine 23-plex assay is semi-quantitative, we more accurately quantified the synergistic interaction of GT, particularly on NO, IL-6 and TNF, using ELISA assays. These results confirmed the synergistic interaction of GT in reducing the levels of NO, IL-6 and TNF, manifested as the lower dosage required for the same level effect by CI analysis. IL-6 and TNF have been recognized as key drivers of cytokine storm and have been extensively studied regarding their essential roles in many inflammatory diseases (Tisoncik et al., 2012). The synergistic effect of GT in reducing these two cytokines suggests its potential to target major inflammatory conditions including cytokine storm.

In addition, we observed that GT effectively reduced several chemokines including GM-CSF. The roles and functions of GM-CSF and G-CSF have been linked closely to autoimmunity (including rheumatoid arthritis) and chronic inflammation (Hamilton, 2002). The depletion of GM-CSF was associated with greater disease suppression in arthritis models compared with TNF deprived controls (Hamilton, 2002). GT showed a significant, synergistic inhibition of GM-CSF, suggesting its potential in attenuating the inflammation related to arthritis and chronic inflammation *via* reducing the GM-CSF pathway. However, GT did not show a strong inhibition of G-CSF which may warrant further investigation. GT also showed synergy and low IC_50_ values (< 15 μg/ml) in inhibiting many other cytokines and chemokines in the cytokine array assay, highlighting its potential therapeutic value in a broad range of inflammatory related pathological conditions. Thus, our results demonstrate a promising synergistic combination that could be applied in the management of various inflammatory diseases

We also have examined the relevant signaling pathways and molecular targets involved in inflammation to explain the observed synergy between G and T. TLRs recognize a variety of pathogen-associated molecular patterns to initiate various signaling pathways leading to inflammation (Kawasaki and Kawai, 2014). TLR activation is one of the first defensive mechanisms against invading pathogens and tissue injury, but the dysregulated TLR signaling could disrupt the immune homeostasis with sustained pro-inflammatory cytokines and chemokines secretion (Zhu et al., 2014). Our results suggest that the inhibitory activities of G, T and GT on LPS-induced inflammation begins with the TLR4 receptor directly, as an early regulatory event of the subsequent inflammatory response. Previous research has demonstrated that 6-shogaol and curcumin, key bioactive compounds from G and T respectively, inhibited the homodimerization of TLR4 (Youn et al., 2006; Ahn et al., 2009) and the expression of TLR4 (Zhou et al., 2015) which may contribute to the effect of G, T and GT on TLR4 in the present study. Thus, GT may serve as a novel TLR inhibitor which possesses the therapeutic potential for modulating innate immune molecular pathways. Our observation that G and T down-regulates the downstream protein targets is in line with previous studies of various forms of ginger and turmeric extracts (Choi et al., 2013; Rana et al., 2015). Our study has further revealed that G and T had similar targets in the TLR4-TRAF6-MAPK pathway, including TLR4, TRAF6 and p-c-JUN. It was not surprising to see the multi-target behavior of G and T as individual extracts, as their bioactive compounds (e.g., 6-gingerol, 6-shogaol, curcumin) have been reported to exhibit broad anti-inflammatory activity *via* various mechanistic pathways (Pan et al., 2008; Jurenka, 2009; Akram et al., 2010; Dugasani et al., 2010; Chen et al., 2019). However, significant inhibition was seen in GT acting on both upstream and downstream protein in this axis, and the combined effect appears to be accumulated from the upstream and increased at the downstream protein targets including p-JNK and p-c-JUN. This is the first analysis of the combined action of G and T on the key proteins in the TLR4-TRAF6-MAPK pathway and demonstrates how synergy occurs in the axis. Network pharmacology analysis might be useful to explain how the compounds in G and T contribute to the strengthened effect of key protein targets in the associated pathways.

Another pathway that may be involved in the synergistic activity of GT is the translocation of NF-kB which plays a central role in inflammatory response in cells ^46^. We found that the GT combination further reduced NF-κB translocation and that this effect was markedly greater than for G or T individually at the same concentration level. Many studies have reported the bioactive components (i.e., 6-gingerol, 6-shogaol) in ginger with anti-inflammatory activity *via* NF- kB (Shim et al., 2011), but the mechanistic studies on ginger as a whole extract are few (Tripathi et al., 2006; Saedisomeolia et al., 2019). The dose-response effect of ginger extract largely depends on the quality and form of ginger (i.e., fresh or dried ginger) which affects the chemical composition and amount of bioactive compounds. One study has linked the inhibitory effect on NF-κB pathway to the therapeutic potential in diabetes, and thus considered ginger as a target agent in the treatment and control of diabetes (Saedisomeolia et al., 2019). On the other hand, although the main bioactive compound of turmeric, curcumin, has been extensively reported to have potent anti-inflammatory activity associated with NF-κB signaling, the effect of T alone was not prominent in our study. In the present study, T was semi-refined with three enriched curcuminoids. If curcumin supposes to have a potent effect against NF-κB location, then there might be antagonistic effects among the three curcuminoids within T that lead to reduced effectiveness of the extract. A recent study by Zarei *et al* (2021) showed that lipid-solubles from ginger and turmeric dissolved in deep-fried vegetable oils attenuated hepatic inflammation in rats, and the individual action was associated with the downregulation of NF-κB (Zarei et al., 2021). This indicates that the further strengthened effect from the combination may be attributed to the actions of the lipid-soluble components from G and T. Further study of the interaction of lipid soluble compounds in G and T is warranted to explain the enhanced activity. Nevertheless, the strengthened reduction of NF-κB translocation by GT could be widely applicable to various pathological conditions relevant to inflammation.

We also examined the synergistic mechanism of G and T *via* regulating the miR-155-5p expression. Upon LPS stimulation, miR-155 is rapidly upregulated by NF-κB within the first 12 h of inflammatory response (Mahesh and Biswas, 2019). The overexpression of miR-155 may potentially regulate many key cytokines (IL-6, IL-1β and IL-8) in the cytokine storm and is thus considered as a novel therapeutic target (Gasparello et al., 2021). Our study revealed that G and T both downregulated the increased fold of miR-155-5p induced by LPS, and this action was strengthened in the synergistic GT combination. Since the modulation of miR-155 is closely related to the NF-κB, the suppression of miR-155p by GT may be attributed to the down-regulation of NF-κB translocation, leading to a negative feedback loop modulating miR-155p expression, and/or the direct effect on miR-155 itself. More work is needed to further elucidate this mechanism.

Taken together, our data clearly demonstrate that the synergistic interaction of G and T may be mediated by changes in the expression of multiple key proteins in the upstream signaling pathway, resulting in downstream effects. The synergistic activity in inhibiting multiple pro-inflammatory cytokines supports the use of GT as a novel therapeutic candidate for a broad application to inflammatory diseases with promoted efficacy.

## Data Availability

The raw data supporting the conclusion of this article will be made available by the authors, without undue reservation.
