# Supplementary material for: Synergistic Inhibition of Pro-Inflammatory Pathways by Ginger and Turmeric Extracts in RAW 264.7 Cells
Source: Front Pharmacol. 2022 May 19;13:818166. doi: 10.3389/fphar.2022.818166 (PMC9160922; doi:10.3389/fphar.2022.818166)
Supplement: Supplementary file 1 [file Image1.pdf]

Supplementary Material

Supplementary Figures

A

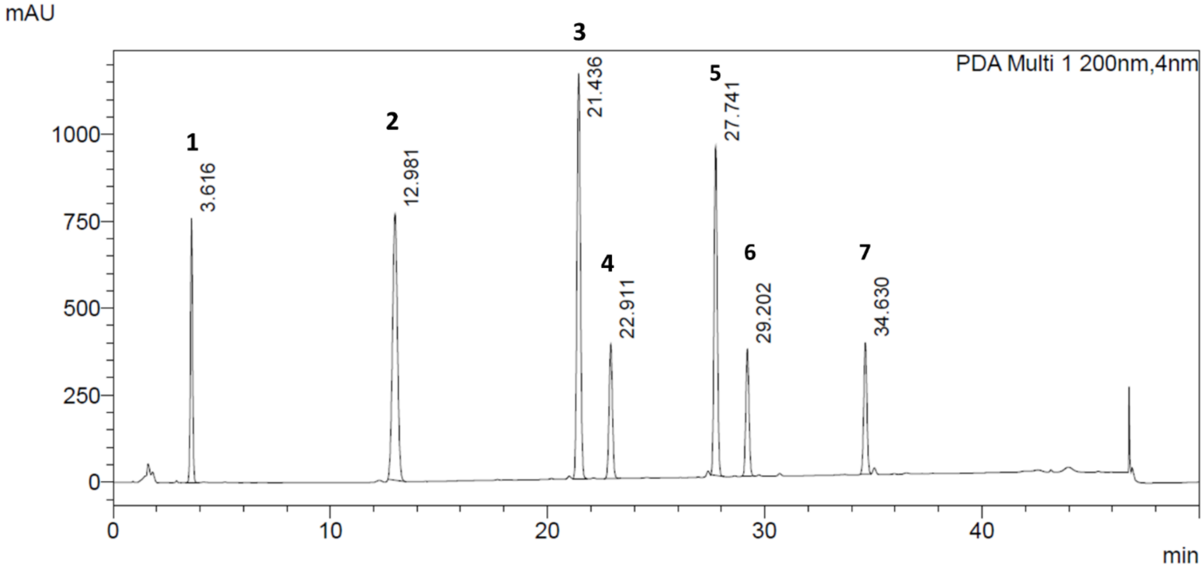

B

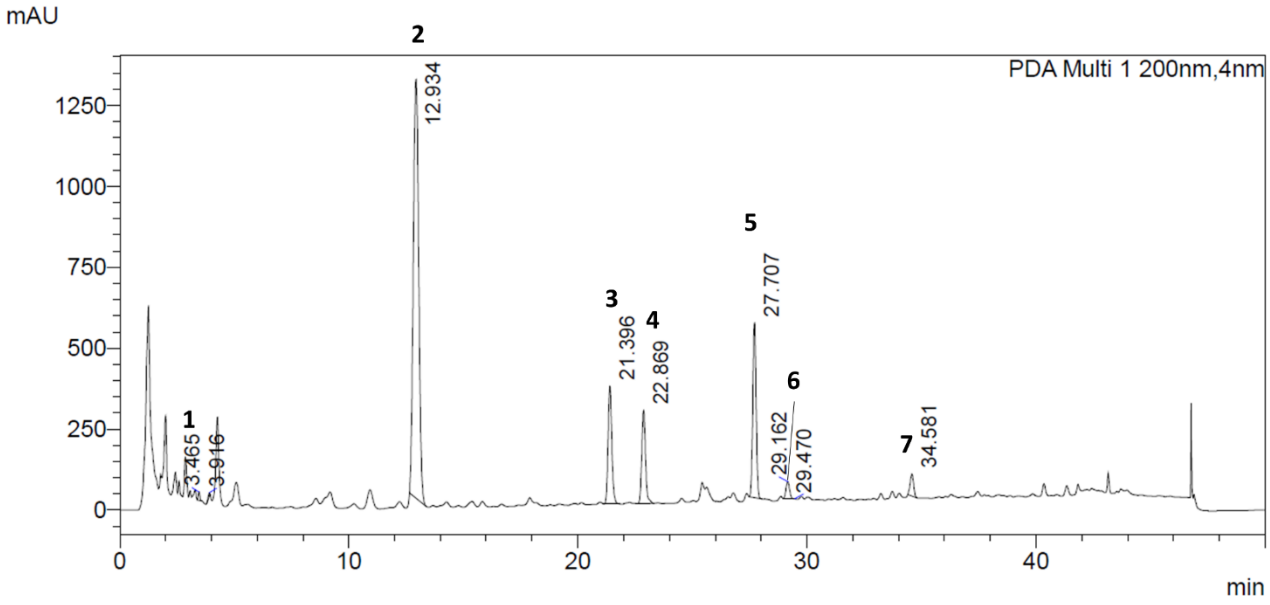

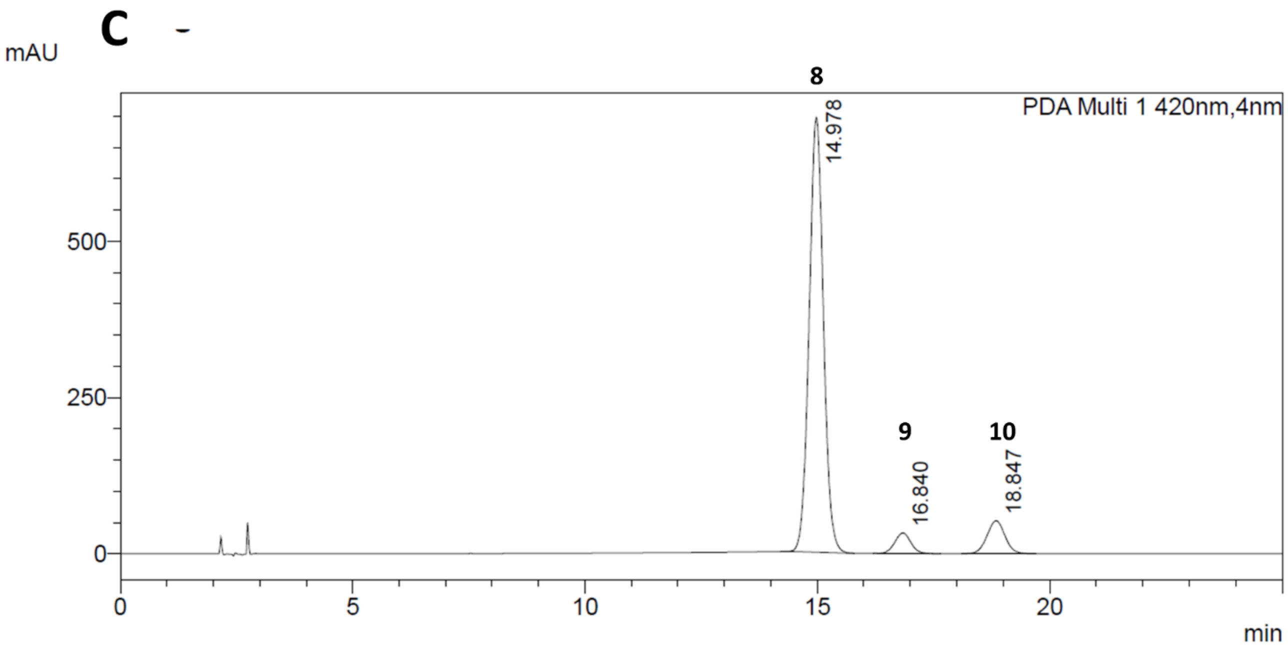

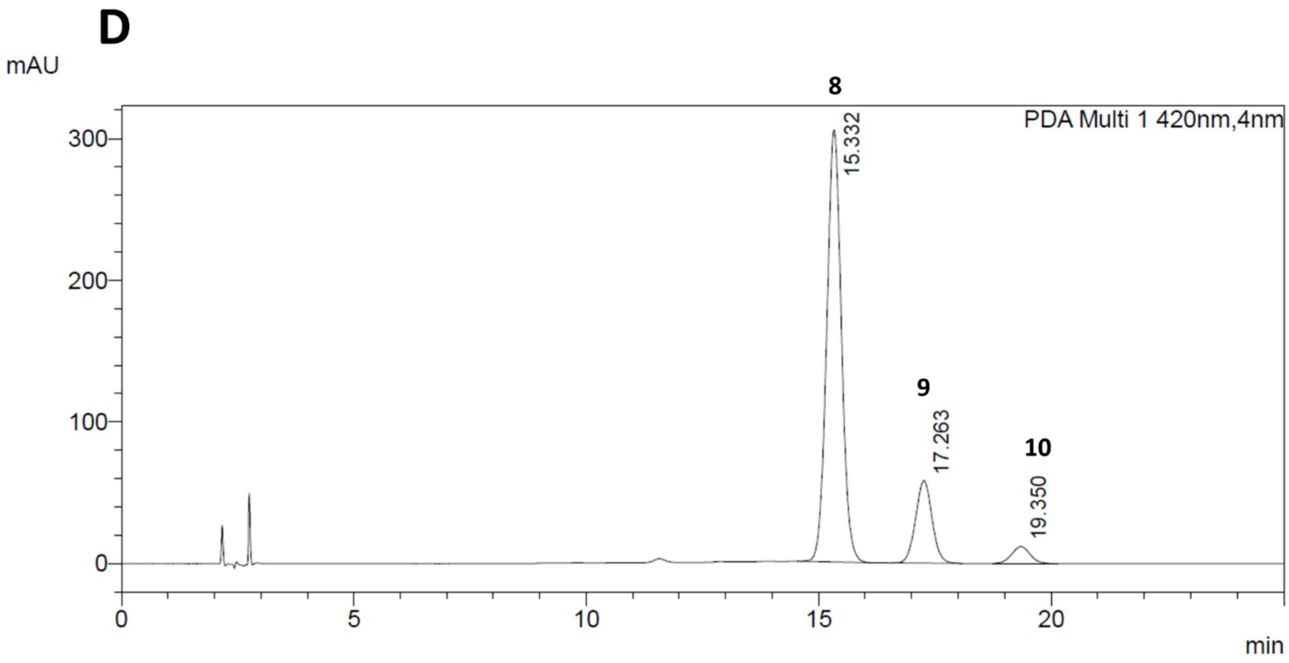

Supplementary Figure 1. HPLC chromatograms of mixed standards and extracts of ginger (A-B) and turmeric (C-D). 1. Zingerol, 2. 6-gingerol, 3. 8-gingerol, 4. 6-shogaol, 5. 10-gingerol, 6. 8-shogaol, 7. 10-shogaol. 8. Curcumin, 9. Demethoxycurcumin, 10. Disdemethoxycurcumin.
